# Neuroprogression across the Early Course of Psychosis

**DOI:** 10.20900/jpbs.20200002

**Published:** 2020-02-11

**Authors:** Kathryn E. Lewandowski, Sylvain Bouix, Dost Ongur, Martha E. Shenton

**Affiliations:** 1 Schizophrenia and Bipolar Disorder Program, McLean Hospital, Belmont, MA 02478, USA; 2 Department of Psychiatry, Harvard Medical School, Boston, MA 02215, USA; 3 Psychiatry Neuroimaging Laboratory, Brigham and Women’s Hospital, Boston, MA, 02215, USA

**Keywords:** first episode, psychosis, imaging, cognition, longitudinal, schizophrenia

## Abstract

Psychotic disorders are severe, debilitating, and even fatal. The development of targeted and effective interventions for psychosis depends upon on clear understanding of the timing and nature of disease progression to target processes amenable to intervention. Strong evidence suggests early and ongoing neuroprogressive changes, but timing and inflection points remain unclear and likely differ across cognitive, clinical, and brain measures. Additionally, granular evidence across modalities is particularly sparse in the “bridging years” between first episode and established illness—years that may be especially critical for improving outcomes and during which interventions may be maximally effective. Our objective is the systematic, multimodal characterization of neuroprogression through the early course of illness in a cross-diagnostic sample of patients with psychosis. We aim to (1) interrogate neurocognition, structural brain measures, and network connectivity at multiple assessments over the first eight years of illness to map neuroprogressive trajectories, and (2) examine trajectories as predictors of clinical and functional outcomes. We will recruit 192 patients with psychosis and 36 healthy controls. Assessments will occur at baseline and 8- and 16-month follow ups using clinical, cognitive, and imaging measures. We will employ an accelerated longitudinal design (ALD), which permits ascertainment of data across a longer timeframe and at more frequent intervals than would be possible in a single cohort longitudinal study. Results from this study are expected to hasten identification of actionable treatment targets that are closely associated with clinical outcomes, and identify subgroups who share common neuroprogressive trajectories toward the development of individualized treatments.

## INTRODUCTION

Psychotic disorders, including schizophrenia (SZ), schizoaffective disorder (SZA), and bipolar disorder with psychosis (BDP) are severe, debilitating, and even fatal [1] and are a leading cause of disability world-wide [2]. Unfortunately, a majority of patients with psychosis experience functional impairments, even after symptom remission [3,4], underscoring further the need for effective, targeted treatments to improve outcomes. The development and implementation of targeted and effective treatments for psychosis is critically dependent on a clear understanding of the timing and nature of disease progression in order to target processes amenable to intervention.

Evidence of early and ongoing disease-related changes in brain and neurocognitive measures in psychosis, often referred to as “neuroprogression,” including cognitive dysfunction, gray matter reduction and ventricular enlargement, and regional structural and connectivity alterations, have been described using multiple modalities including neurocognitive testing, PET, CT, and fMRI imaging techniques, and post-mortem brain studies (Table 1) [5–93]. However, these studies rely mainly on cross-sectional data comparing groups at particular illness stages (e.g., high risk, first episode, established) to healthy controls or to each other. Existing longitudinal studies typically involve only two measurement points, or repeated assessment years apart, thereby limiting our understanding of the actual trajectories and key inflection points of these disease markers [94]. Thus, while it is evident that significant progressive brain changes occur during the years following an initial episode, the timing and course of progression across domains remains unclear. This knowledge gap limits our ability to develop interventions that capitalize on plasticity in key systems during this critical and dynamic period of illness, and hinders the development of targeted treatments when they may be most effective, potentially preventing further decline and chronic loss of functioning.

### Early Psychosis: A Critical Period

The years after illness onset represent a critical period where early intervention strategies may be most effective, before irreversible brain alterations take place. Effective treatment after a first episode of psychosis (FEP) not only improves functioning but may actually alter illness trajectories placing patients on a path toward recovery [95]. Disease trajectories appear to crystalize in the years following an initial episode of illness, making this a critical period for intervention after which effectiveness may be greatly reduced [96,97]. While much is known about neuroprogressive changes in FEP and established illness, less is known about the course and timing of these changes in the “bridging years” between illness stages. The National Advisory Mental Health Council’s Workgroup Report recommended that in the identification of pathophysiological processes that contribute to symptoms or syndromes “[p]articular attention should be devoted to discovering the sensitive and critical periods when neuroplasticity in specific circuits is greatest and maximally responsive to intervention” [98]. This requires careful phenotyping of neuroprogression throughout the early course of illness and development of predictive models.

### Neuroprogression in Early Psychosis

Abnormalities of gray and white matter volume, network connectivity, and cognition are well-described in patients with established psychosis, and longitudinal research suggests that neuroprogression may continue well into chronicity in some patients [65,67,99]. However, neuroprogressive changes may begin much earlier in the illness course, some even prior to illness onset. Examination of cognition, gray matter, white matter, and connectivity at various stages of illness suggest that (1) measurable alterations exist in each of these domains and (2) abnormalities do not progress uniformly across stages of illness (Table 1). For instance, cognitive abnormalities appear to be present prior to illness onset in patients with SZ, becoming more widespread by the first episode with profiles qualitatively and perhaps quantitatively similar to chronicity in both BP and SZ [26]. In contrast, while gray matter (GM) and white matter (WM) reductions are reported in multiple frontal, temporal, and parietal regions by the first episode, and these markers appear to become more widespread and pronounced compared to controls into chronicity. Regionally, some brain volume abnormalities appear to be present in first episode at the magnitude seen in chronicity (e.g., hippocampal volume), whereas some structures (e.g., amygdala) that show significant abnormalities in established psychosis show no evidence of abnormalities in high risk [59,100]. Thus, neuroprogression is detectable early in the course of illness; however, neuroprogressive changes across modalities do not occur uniformly.

### Neuroprogression and Illness Course

Neuroprogressive changes are related to disease course. Cognitive deficits are predictive of functional disability [3,24], and progressive gray matter loss and increased cerebrospinal fluid volumes are associated with symptom severity, clinical course, and poorer functional outcomes [48,65,101–103]. Associations amongst cognitive and brain measures suggest complex dynamics amongst these domains, and with illness course and functional outcomes [30,58,64,104–106]. For instance, in a recent study functionally connected brain regions “thinned together” in networks related to cognition, showing a dynamic interplay amongst cognitive, structural, and connectivity changes [107].

Together, findings suggest that neuroprogressive changes in the years following psychosis onset occur rapidly in many areas during an important neurodevelopmental window [94], with continued progression throughout the early and mid years. However, the current state of the literature is insufficient to carefully characterize neuroprogressive trajectories at key inflection points [29] and throughout the “bridging” years between onset and established illness—years that are critical for targeted intervention—for several reasons. First, studies of high risk participants often have very low rates of conversion to psychosis resulting in possible “dilution effects” [94,108,109]. Second, most longitudinal studies include only two assessment points forcing the assumption of linearity, and those with more assessments are typically years apart making it difficult to pinpoint inflection points in primary outcomes [94]. It has been suggested that repeated assessments (>2) at relatively short (i.e., at most 1 year apart) intervals are needed to characterize the longitudinal trajectories in each of these neuroprogressive domains [94,110]. Other challenges include considerable methodological variation across studies (e.g., definition of key grouping characteristics (e.g., DOI in first episode studies); inter-scan interval; analysis approach). Additionally, as can been seen in Table 1, relatively few studies focus explicitly on the years between onset and chronicity, despite the critical nature of the early course of illness in terms of prognosis and intervention.

### Heterogeneity in Cognition and Neurobiology

Heterogeneity of premorbid adjustment, illness course, and outcomes is the rule rather than the exception in psychotic disorders, (e.g., [62]), and identifiable subsamples may differ in neuroprogressive degree and trajectory (e.g., [111]). Recent reports suggest that abnormalities in brain structure and connectivity are associated with cognitive subtypes in psychosis [112–114]. A recent study found that baseline neurocognitive functioning predicted gray matter reductions in multiple brain regions two years later [115], suggesting that baseline profiles may predict neuroprogressive course. While heterogeneity can interfere with our ability to identify associations and timelines at the group level, it may be possible to leverage this heterogeneity to identify subgroups that share similar behavioral and neurobiological presentation and course toward more individualized prediction and treatment implementation.

### Goals and Hypotheses

Evidence strongly indicates that neuroprogressive changes occur across brain and cognitive measures in patients with psychosis at the time of first episode and throughout the early course of illness. Nonetheless, no studies to date have undertaken the systematic characterization of neuroprogression throughout the early course of illness at a granular level using multimodal assessments in a transdiagnostic sample. Thus, our objective with this project is the systematic, multimodal characterization of neuroprogression through the early years of illness in a cross-diagnostic sample of patients with psychosis. We hypothesize that (1) by interrogating neurocognition, structural brain measures, and network connectivity at multiple assessments across the first eight years of illness we will identify clear neuroprogressive trajectories along our primary outcome variables, and (2) neuroprogressive trajectories will be predictive of clinical and functional outcomes. To accomplish this objective within the project timeline, we will use an accelerated longitudinal design (ALD, described below) modeling multiple neuroprogressive markers by duration of illness, and in association with key clinical and functional measures. A central aspect of this project is that it builds upon the rich neuroimaging, cognitive, and clinical data being collected by the Human Connectome Project in Early Psychosis (HCP-EP; PI: Dr. Martha Shenton). Data collection for this longitudinal study will occur across the Boston HCP-EP sites, and it is estimated that approximately 75% of baseline data for the present project will be drawn from existing data collected in the context of the HCP-EP.

## MATERIALS AND METHODS

### Participants

192 patients with DSM-V non-affective (schizophrenia, schizophreniform, schizoaffective, psychosis NOS, delusional disorder, brief psychotic disorder) or affective (major depression with psychosis or bipolar disorder with psychosis) psychosis as determined by SCID-5-RV for DSM-V-RV interview [116] will be enrolled. As noted above, it is expected that approximately 75% of these subjects will have participated previously in the HCP-EP. Patients will be between the ages 18–35 at enrollment, and between the ages of 17 and 30 at the time of their first episode. Duration of illness will be determined via the SCID interview, together with all available collateral data from medical records, treatment providers, and family members. Subjects must have capacity to provide informed consent. Exclusion criteria include MRI contraindication, IQ less than 70 based on medical history or WASI-II [117], DSM-V diagnosis of substance-induced psychosis or psychotic disorder due to medical condition [116] and known brain damage. We will also recruit 36 control participants. Exclusion criteria for control participants include history of DSM-V diagnosis or psychiatric treatment, and all other exclusion criteria noted above. All procedures have been approved by the Partners Healthcare Human Research Committee/IRB, and comply with the regulations set forth by the Declaration of Helsinki.

### Materials

In order to capitalize on existing data and maximize comparability between data sets, we will use identical materials and procedures to those currently implemented by the HCP-EP. Participants will be reassessed at the same site as their original assessment, including MRI scans.

#### Behavioral measures

Behavioral measures include the NIH Cognition Toolbox [118], psychosis-relevant HCP Lifespan measures, and additional measures for early psychosis including: (1) Hollingshead Two-Factor scale [119], measure of parental SES; (2) SCID-5-RV in conjunction with medical records and patient/family clinical interviews to confirm diagnosis; (3) The Positive and Negative Syndrome Scale (PANSS) [120]; (4) The Clinical Assessment Interview for Negative Symptoms (CAINS) [121]; (5) The Young Mania Rating Scale (YMRS) [122]; (6) The Montgomery-Asberg Depression Rating Scale (MADRS) [123]; (7) The MIRECC Global Assessment of Functioning (GAF) [124]; (8) HCP-EP Lifetime Medication Record, which assesses past and current medication use; 9) WASI-II Vocabulary and Matrix Reasoning to estimate IQ, and 10) the Seidman Auditory Continuous Performance Test (CPT [125,126]).

Medications represent an important covariate in the study of progressive changes in brain and behavior. We will collect detailed information about mediation use at each assessment, and account for medication effects using analytic models of antipsychotic, Lithium, and total medication load both as moderators and confounders in order to examine the potential role of medications in neuroprogression.

#### Neuroimaging: MR data acquisition protocol

Imaging data will be collected on two Siemens MAGNETOM Prisma 3T scanners, one at McLean and one at Brigham and Women’s Hospital. Both use a 32-channel head coil and are actively collecting HCP-EP data using the same sequence employed here. This protocol is similar to the original HCP Lifespan protocol [127], but without the fMRI task, as many subjects with psychotic disorders may not easily tolerate lengthy MR sessions. Total scan time is just over one hour. The scan sequences include: (1) Localizer and Auto Align Scout; (2) Structural T1w (MPRAGE) (0.8 mm isotropic; T1 1000 ms; TR 2400 ms; 208 slices) and T2w (SPACE) (0.8 mm isotropic; TR 3200 ms; 208 slices) (3) Resting state fMRI (rfMRI) of 2mm isotropic; multiband (MB) acceleration × 8; TR 720 ms; acquired twice: once with AP and once with PA phase encoding; 4) Diffusion MRI (dMRI) 1.5 mm isotropic; TR: 3230 ms; TE: 89.20 m; flip angle 78°; MB acceleration × 4, 92 directions in each shell (b = 1500 and 3000) acquired twice: once with AP and once with PA phase encoding. Field maps will be acquired to correct for intensity and geometric distortions.

#### Procedures

This project employs an accelerated longitudinal design (ALD) in order to cover the desired timeframe within the constraints of the project. ALDs follow enrollment cohorts longitudinally and thus model both within subject longitudinal and between subject group effects. While most ALDs use age to define cohorts, we will use duration of illness (DOI), which will allow us to estimate DOI-related trends in our primary outcomes. The timing of repeated assessments will be calibrated to each individual’s DOI resulting in measures that span the range from 0 to 88 months after illness onset. We selected a “balanced ALD”, meaning equally spaced measurements across the study, the same number of measurements per cohort, and equal overlap between successive cohorts (in this case, no overlap; see Table 2).

#### Data collection schedule

Assessments will be conducted at baseline, 8-months and 16-months to fully cover the early course of illness (Table 2) at equally-spaced assessments, minimizing overlap of assessment points amongst cohorts (desirable in an ALD [128]). Baseline assessments include MRI scan, clinical and diagnostic interviews, and neuropsychological assessments. Follow up at 8- and 16-months will consist of the MRI scan, clinical and neuropsychological assessments. The follow up interval was selected because scans at least annually have been recommended for assessment of changes that occur relatively rapidly (e.g., [20,94]) as may be the case after psychosis onset [62], and coverage of the time between onset and established illness is needed to fill a critical knowledge gap. Thus, in the context of an ALD, assessments at 8-month intervals permits frequent assessments at multiple assessment points—important for assessment of trajectories without forcing an assumption of linearity [20,94]—while covering nearly 8 years after illness onset. Control participants will also be assessed three times at baseline, 8- and 16-months.

#### Behavioral assessments

Follow-up assessments involve approximately 3–3.5 h of behavioral, clinical, cognitive testing. Baseline assessments will also include a clinical diagnostic interview that will add approximately 1.5–2 h. Total assessment time for baseline procedures is expected to take approximately 4.5–5 h over two or more days, within days of the imaging. Participants are provided lunch and/or snacks during the assessment, as appropriate. Reliability was established on all Toolbox measures prior to the start of enrollment. Reliability and consensus diagnosis is ongoing for all diagnostic interview, conducted on a monthly basis with all diagnostic team staff across sites.

#### MRI scans

Subjects complete MRI safety screening prior to scanning. Procedures are described to subjects and they are helped into the scanner by study staff and an MRI tech at the scanning site. Subjects are instructed to remain still during scanning and deformable foam cushioning is used to stabilize the head. Real time image reconstruction and processing are used for quality assurance at the time of scanning. Total scan time is just over 1 h; with MRI safety checks total time at the scan site is approximately 1.5 h.

All MRI data processing and storage are completed via a central database system at Brigham and Women’s hospital, which has been customized to host the project data and to manage daily operations and QC procedures and synchronized to receive data directly from McLean. This upload tool automatically strips all PHI prior to upload, and de-identified data from both sites with QC are stored in this database system. Automated verification of scan acquisition parameters at the time of the scan are followed by a manual review, and a semi-automated QC procedure developed to detect signal drops is run for each scan. Because two different scanning sites will acquire data, special considerations have been taken to ensure that the data quality is homogeneous across sites. Harmonization procedures include Siemens specific QA tools, phantom measurements (fBIRN and NIST phantoms), and traveling human subjects. Scanner reliability was assessed both between scanners and using test-retest assessments within scanners. Intraclass correlations (ICC) were computed on Freesurfer outputs including total GM volume, subcortical GM volume, cortical WM volume, brain segmentation volume, and total ICV, and regionally specific measures based on our primary outcomes. We found ICCs of 0.98–0.99 for broad measures, and 0.90–0.99 for regional measures.

#### Planned analyses

Our primary aim is to map neuroprogression across the first 8 years of illness. We will fit latent growth curve models to the repeated measures of our cognitive and MRI measures on the full sample using techniques that permit the functional form of the trajectories to be determined by the data. Each model will include initial DOI as a covariate to separate between-subject differences from within-subject changes as a function of current DOI at each assessment point [129,130]; the model will also include age at assessment to evaluate the effects of natural aging, which will also be estimated from the control participant data, as well as demographic and clinical covariates. By including the between-subjects and within-subjects separately in the models we are able to determine the extent to which trajectories are more strongly associated with longitudinal change over DOI, or cohort effects. These methods allow examination of inflection points in primary outcome trajectories, and peaks and valleys by DOI. We will also examine diagnostic differences in trajectories.

To evaluate the predictive utility of neuroprogressive change on clinical and community functioning, we will conduct a second set of latent growth curve analyses on clinical and functional measures. We will then build predictive models of our clinical and functional outcomes based on within-subject changes in neuroprogressive markers. We will also explore the possibility that subgroups of subjects demonstrate distinct neuroprogressive trajectories, and differences in clinical and functional outcomes based on these groups using growth mixture models (GMMs). GMMs, an extension of multiple-group growth curve models in which the grouping variable is not specified a priori, can be used to identify subgroups within the data and describe differences in longitudinal trajectories between subsamples. Finally, we will conduct explicit tests of fully dimensional models and combined dimensional and categorical models based on the latent class trajectories groupings.

## DISCUSSION

Determining the timing and course of neuroprogressive changes over the early course of psychosis is essential to the development and implementation of targeted, individualized treatment during a critical time period in which treatments may be maximally effective and the potential for preventing further decline and chronic loss of functioning is at its greatest. Results from this study will (1) hasten the identification of actionable treatment targets that are closely associated with clinical outcomes in order to capitalize on islands of preserved plasticity and maximize their clinical utility, and (2) provide guidance for individualized treatment.

This project includes several key innovations. First, these data will be the first to characterize multiple markers of neuroprogression throughout the early course of psychosis, including inflection points, stabilization points, and associations with clinical course, in a transdiagnostic sample and within a single study paradigm covering the critical years between first episode and established illness. These findings will not only elucidate neuroprogressive processes that are as yet unknown, but will hasten our ability to design treatments in the early course of illness that target actionable mechanisms, potentially preventing further decline and chronic loss of functioning. For instance, if neurocognitive decline predates and predicts structural brain degeneration in associated regions, early treatments targeting cognition for patients with cognitive deficits may improve cognition and halt progression of gray matter loss. Indeed, Eack et al. [131] found that cognitive remediation both improved cognition and slowed gray matter loss in patients with SZ. Additionally, we will examine heterogeneity of neuroprogressive trajectories and their associations with clinical and functional course. The use of an accelerated longitudinal design (ALD) will allow ascertainment of data across a longer timeframe than would be possible in a single cohort longitudinal study, and at more frequent intervals than may be feasible in the same subjects over eight years [128]. To our knowledge this is the first study to employ an ALD based on illness duration in early psychosis. Of course, the use of an ALD rather than a fully longitudinal design in a single cohort introduces the possibility of cohort effects, but we feel that the benefits outweigh the costs by permitting coverage of the full 8 years of interest while reducing the likelihood of large-scale attrition over such a long follow up thereby reducing power especially in the later years, frequent enough inter-assessment intervals to capture inflection and stabilization points in a more fine-grained way, and the potential to fit non-linear models by including 3 assessments per participant. Additionally, we are in a unique position to leverage a major ongoing study of patients in early psychosis to achieve our aims, capitalizing on both the innovations of the HCP-EP and the timing of the study. The HCP-EP is currently enrolling, allowing us to incorporate longitudinal data to the project while data collection is ongoing, and existing data will serve as baseline assessments for a large proportion of the sample allowing us to increase overall enrollment and follow-up and thereby increase power adequately to perform the analyses described above.

## Figures and Tables

**Table 1.** Neuroprogressive Alterations across Illness Stages.

Domain	High Risk	First Episode	Early Course	Chronicity
**Cognition**	SZ- Attenuated or selective deficits relative to chronic patients [5–8] BD- no evidence of premorbid deficits; relatives show verbal memory and executive function deficits [9–14]	Cognitive impairment in SZ and BD [9,11,15–19] Some reports of widespread impairment, others of more selective impairments [20]	Mixed findings: some estimate no additional decline during the early course [21–23]; some show continued decline [24,25], and some show improvement in some areas [23]	Widespread deficits: BD- ~1 SD below the mean [26–28]; SZ- ~2 SD below the mean [3,11,29–31] May be associated with relapse or symptom severity [25,32,33] but findings mixed [22,26]
**Structural**	GM reductions in middle frontal, prefrontal, superior temporal, ACC, thalamus, hippocampus, parahippocampus [5,34,35] *Longitudinal*: volume reductions, thinning, SA contraction in whole brain, frontal, superior temporal, fusiform and insula, ACC, precuneus, parahippocampus, ventricular enlargement [36–43] *Converters* vs *Non*: reduced insula, ACC, callosum, temporal lobe; increased gyrification [5,40,41,44–47]	Reductions in whole brain, superior temporal, medial frontal, prefrontal and ACC cortices, cerebellum, insula, amygdala, caudate, and ventricular enlargement [5,34,35,48–58] Hippocampal reductions may be comparable to chronic patients [34,52,59–61] *Longitudinal*: Progressive reductions in cortical SA, whole brain, frontal, temporal, parietal, limbic regions over short time (~2 years) [62]	Frontal, temporal and parietal GM, thalamic volume loss [62,63], most pronounced in the first 2 years after baseline [62] Reduced volume and thickness in multiple regions, which was associated with age [64] Associated with cognitive impairment but only weakly with clinical measures [62]	Significant, widespread GM reductions, including whole brain volume, medial and superior temporal, inferior parietal, frontal, occipital, ACC, hippocampus, parahippocampus, amygdala, insula, thalamus, and cerebellum and ventricular enlargement [34,45,48,49,58–60,65–75] *Longitudinal*: progressive volume loss into chronicity; greatest annual reduction in superior temporal regions [67]
**Connectivity**	Reduced in salience, control, auditory and motor networks [76,77]	Mixed findings: Reduced in frontal lobes [78]; abnormalities in DMN and Control Networks [78–80] No difference from HC [81] Improved over 1 year with clinical improvement [82]		More pronounced and extended frontal, temporal and sensorimotor abnormalities [78,81] BD and SZ show similar within network reductions in DMN, Control and Visual networks [83]
**White Matter**	Mixed: increased in regions of the frontal lobes; decreased in medial temporal and superior parietal, corpus callosum [51,84,85] *Longitudinal*: reduced in fronto-occipatal fasiculus and cerebellar-thalamic regions during transition [85,86]	Reduced FA in corpus callosum, internal capsule, external capsule, fornix, superior, temporal, inferior fronto-occipital fasciculus, cingulum, uncinate fasciculus; widespread increased diffusivity [87–89] Abnormalities in thalamo-cortical WM connectivity [90]	Frontal and temporal WM volume reductions [62] WM reductions, which were associated with age [64,91]	Widespread prefrontal and frontal, temporal, internal capsule WM reductions [87,91,92] BD and SZ show abnormalities unrelated to age or DOI) [93]

SZ: Schizophrenia; BD: Bipolar Disorder; SD: standard deviation; GM: gray matter; ACC: anterior cingulate cortex; SA: surface area; DMN: Default mode network; HC: healthy control; WM: white matter; DOI: duration of illness.

**Table 2.** Assessment Schedule by Cohort.

Months Since Onset	0	8	16	24 (2 year)	32	40	48 (4 year)	56	64	72 (6 year)	80	88
**Cohort 1 (0 years)**	×	×	×									
**Cohort 2 (2 years)**				×	×	×						
**Cohort 3 (4 years)**							×	×	×			
**Cohort 4 (6 years)**										×	×	×

Assessment schedule by time (months since onset) and duration of illness-defined cohort.

## References

[1] Kessler RC, Chiu WT, Demler O, Merikangas KR, Walters EE. Prevalence, severity, and comorbidity of 12-month DSM-IV disorders in the National Comorbidity Survey Replication. Arch Gen Psychiatry. 2005;62:617–27. 15939839 10.1001/archpsyc.62.6.617 PMC2847357

[2] WHO. Global Burden of Disease. Available from: https://www.who.int/healthinfo/global_burden_disease/about/en/. Accessed 2020 Feb 7.

[3] Green MF. Cognitive impairment and functional outcome in schizophrenia and bipolar disorder. J Clin Psychiatry. 2006;67(Suppl 9:3–8); discussion 36–42. 16965182

[4] Barch DM. Neuropsychological abnormalities in schizophrenia and major mood disorders: similarities and differences. Curr Psychiatry Rep. 2009;11:313–9. 19635240 10.1007/s11920-009-0045-6 PMC3836606

[5] Johnstone EC, Lawrie SM, Cosway R. What does the Edinburgh high-risk study tell us about schizophrenia. Am J Med Genet. 2002;114:906–12. 12457384 10.1002/ajmg.b.10304

[6] Carrión RE, Walder DJ, Auther AM, McLaughlin D, Zyla HO, Adelsheim S, From the psychosis prodrome to the first-episode of psychosis: No evidence of a cognitive decline. J Psychiatr Res. 2018;96:231–8. 29121595 10.1016/j.jpsychires.2017.10.014 PMC7663810

[7] Fuller R, Nopoulos P, Arndt S, O’Leary D, Ho BC, Andreasen NC. Longitudinal assessment of premorbid cognitive functioning in patients with schizophrenia through examination of standardized scholastic test performance. Am J Psychiatry. 2002;159:1183–9. 12091197 10.1176/appi.ajp.159.7.1183

[8] Bilder RM, Reiter G, Bates J, Lencz T, Szeszko P, Goldman RS, Cognitive development in schizophrenia: follow-back from the first episode. J Clin Exp Neuropsychol. 2006;28:270–82. 16484098 10.1080/13803390500360554

[9] Olvet DM, Burdick KE, Cornblatt BA. Assessing the potential to use neurocognition to predict who is at risk for developing bipolar disorder: a review of the literature. Cogn Neuropsychiatry. 2013;18:129–45. 23137046 10.1080/13546805.2012.724193 PMC3578087

[10] Calafiore D, Rossell SL, Van Rheenen TE. Cognitive abilities in first-degree relatives of individuals with bipolar disorder. J Affect Disord. 2018;225:147–52. 28829959 10.1016/j.jad.2017.08.029

[11] Lewandowski KE, Cohen BM, Ongur D. Evolution of neuropsychological dysfunction during the course of schizophrenia and bipolar disorder. Psychol Med. 2011;41:225–41. 20836900 10.1017/S0033291710001042

[12] Seidman LJ, Giuliano AJ, Smith CW, Stone WS, Glatt SJ, Meyer E, Neuropsychological functioning in adolescents and young adults at genetic risk for schizophrenia and affective psychoses: results from the Harvard and Hillside Adolescent High Risk Studies. Schizophr Bull. 2006;32:507–24. 16707777 10.1093/schbul/sbj078 PMC2632246

[13] Keshavan MS, Kulkarni S, Bhojraj T, Francis A, Diwadkar V, Montrose DM, Premorbid cognitive deficits in young relatives of schizophrenia patients. Front Hum Neurosci. 2010;3:62. 20300465 10.3389/neuro.09.062.2009 PMC2839849

[14] Woodberry KA, McFarlane WR, Giuliano AJ, Verdi MB, Cook WL, Faraone SV, Change in neuropsychological functioning over one year in youth at clinical high risk for psychosis. Schizophr Res. 2013;146:87–94. 23434505 10.1016/j.schres.2013.01.017 PMC3633465

[15] Seidman LJ, Giuliano AJ, Meyer EC, Addington J, Cadenhead KS, Cannon TD, Neuropsychology of the prodrome to psychosis in the NAPLS consortium: relationship to family history and conversion to psychosis. Arch Gen Psychiatry. 2010;67:578–88. 20530007 10.1001/archgenpsychiatry.2010.66 PMC3332118

[16] Bora E, Pantelis C. Meta-analysis of Cognitive Impairment in First-Episode Bipolar Disorder: Comparison With First-Episode Schizophrenia and Healthy Controls. Schizophr Bull. 2015;41:1095–104. 25616505 10.1093/schbul/sbu198 PMC4535631

[17] Mesholam-Gately RI, Giuliano AJ, Goff KP, Faraone SV, Seidman LJ. Neurocognition in first-episode schizophrenia: a meta-analytic review. Neuropsychology. 2009;23:315–36. 19413446 10.1037/a0014708

[18] Gruber SA, Rosso IM, Yurgelun-Todd D. Neuropsychological performance predicts clinical recovery in bipolar patients. J Affect Disord. 2008;105:253–60. 17524493 10.1016/j.jad.2007.04.014 PMC3271707

[19] Nehra R, Chakrabarti S, Pradhan BK, Khehra N. Comparison of cognitive functions between first- and multi-episode bipolar affective disorders. J Affect Disord. 2006;93:185–92. 16678909 10.1016/j.jad.2006.03.013

[20] Pantelis C, Wood SJ, Proffitt TM, Testa R, Mahony K, Brewer WJ, Attentional set-shifting ability in first-episode and established schizophrenia: Relationship to working memory. Schizophr Res. 2009;112:104–13. 19464854 10.1016/j.schres.2009.03.039

[21] Rund BR, Barder HE, Evensen J, Haahr U, ten Velden Hegelstad W, Joa I, Neurocognition and Duration of Psychosis: A 10-year Follow-up of First-Episode Patients. Schizophr Bull. 2016;42:87–95. 26101305 10.1093/schbul/sbv083 PMC4681546

[22] Hoff AL, Svetina C, Shields G, Stewart J, DeLisi LE. Ten year longitudinal study of neuropsychological functioning subsequent to a first episode of schizophrenia. Schizophr Res. 2005;78:27–34. 15964177 10.1016/j.schres.2005.05.010

[23] Gold S, Arndt S, Nopoulos P, O’Leary DS, Andreasen NC. Longitudinal study of cognitive function in first-episode and recent-onset schizophrenia. Am J Psychiatry. 1999;156:1342–8. 10484943 10.1176/ajp.156.9.1342

[24] Barder HE, Sundet K, Rund BR, Evensen J, Haahr U, Ten Velden Hegelstad W, Neurocognitive development in first episode psychosis 5 years follow-up: associations between illness severity and cognitive course. Schizophr Res. 2013;149:63–9. 23810121 10.1016/j.schres.2013.06.016

[25] Pukrop R, Schultze-Lutter F, Ruhrmann S, Brockhaus-Dumke A, Tendolkar I, Bechdolf A, Neurocognitive functioning in subjects at risk for a first episode of psychosis compared with first- and multiple-episode schizophrenia. J Clin Exp Neuropsychol. 2006;28:1388–407. 17050266 10.1080/13803390500434425

[26] Bora E, Özerdem A. Meta-analysis of longitudinal studies of cognition in bipolar disorder: comparison with healthy controls and schizophrenia. Psychol Med. 2017;47:2753–66. 28585513 10.1017/S0033291717001490

[27] Burdick KE, Goldberg TE, Cornblatt BA, Keefe RS, Gopin CB, Derosse P, The MATRICS consensus cognitive battery in patients with bipolar I disorder. Neuropsychopharmacology. 2011;36:1587–1592. 21451499 10.1038/npp.2011.36 PMC3138672

[28] Sperry SH, O’Connor LK, Öngür D, Cohen BM, Keshavan MS, Lewandowski KE. Measuring Cognition in Bipolar Disorder with Psychosis Using the MATRICS Consensus Cognitive Battery. J Int Neuropsychol Soc. 2015;21:468–72. 26154947 10.1017/S1355617715000442

[29] Seidman LJ, Buka SL, Goldstein JM, Tsuang MT. Intellectual decline in schizophrenia: evidence from a prospective birth cohort 28 year follow-up study. J Clin Exp Neuropsychol. 2006;28:225–42. 16484095 10.1080/13803390500360471

[30] Dickinson D, Ragland JD, Gold JM, Gur RC. General and specific cognitive deficits in schizophrenia: Goliath defeats David. Biol Psychiatry. 2008;64:823–7. 18472089 10.1016/j.biopsych.2008.04.005 PMC4332801

[31] Dickinson D, Iannone VN, Wilk CM, Gold JM. General and specific cognitive deficits in schizophrenia. Biol Psychiatry. 2004;55:826–33. 15050864 10.1016/j.biopsych.2003.12.010

[32] Robinson LJ, Ferrier IN. Evolution of cognitive impairment in bipolar disorder: a systematic review of cross-sectional evidence. Bipolar Disord. 2006;8:103–16. 16542180 10.1111/j.1399-5618.2006.00277.x

[33] Zubieta JK, Huguelet P, Lajiness-O’Neill R, Giordani BJ. Cognitive function in euthymic bipolar I disorder. Psychiatry Res. 2001;102:9–20. 11368835 10.1016/s0165-1781(01)00242-6

[34] Sprooten E, Papmeyer M, Smyth AM, Vincenz D, Honold S, Conlon GA, Cortical thickness in first-episode schizophrenia patients and individuals at high familial risk: a cross-sectional comparison. Schizophr Res. 2013;151:259–64. 24120958 10.1016/j.schres.2013.09.024

[35] Fusar-Poli P, Radua J, McGuire P, Borgwardt S. Neuroanatomical maps of psychosis onset: voxel-wise meta-analysis of antipsychotic-naive VBM studies. Schizophr Bull. 2012;38:1297–307. 22080494 10.1093/schbul/sbr134 PMC3494061

[36] Sun D, Phillips L, Velakoulis D, Yung A, McGorry PD, Wood SJ, Progressive brain structural changes mapped as psychosis develops in ‘at risk’ individuals. Schizophr Res. 2009;108:85–92. 19138834 10.1016/j.schres.2008.11.026 PMC2670732

[37] Takahashi T, Wood SJ, Yung AR, Phillips LJ, Soulsby B, McGorry PD, Insular cortex gray matter changes in individuals at ultra-high-risk of developing psychosis. Schizophr Res. 2009;111:94–102. 19349150 10.1016/j.schres.2009.03.024

[38] Ziermans TB, Schothorst PF, Schnack HG, Koolschijn PC, Kahn RS, van Engeland H, Progressive structural brain changes during development of psychosis. Schizophr Bull. 2012;38:519–30. 20929968 10.1093/schbul/sbq113 PMC3329986

[39] McIntosh AM, Owens DC, Moorhead WJ, Whalley HC, Stanfield AC, Hall J, Longitudinal volume reductions in people at high genetic risk of schizophrenia as they develop psychosis. Biol Psychiatry. 2011;69:953–58. 21168123 10.1016/j.biopsych.2010.11.003

[40] Pantelis C, Velakoulis D, McGorry PD, Wood SJ, Suckling J, Phillips LJ, Neuroanatomical abnormalities before and after onset of psychosis: a cross-sectional and longitudinal MRI comparison. Lancet. 2003;361:281–88. 12559861 10.1016/S0140-6736(03)12323-9

[41] Takahashi T, Wood SJ, Yung AR, Soulsby B, McGorry PD, Suzuki M, Progressive gray matter reduction of the superior temporal gyrus during transition to psychosis. Arch Gen Psychiatry. 2009;66:366–76. 19349306 10.1001/archgenpsychiatry.2009.12

[42] Takahashi T, Wood SJ, Soulsby B, McGorry PD, Tanino R, Suzuki M, Follow-up MRI study of the insular cortex in first-episode psychosis and chronic schizophrenia. Schizophr Res. 2009;108:49–56. 19171466 10.1016/j.schres.2008.12.029

[43] Borgwardt SJ, Riecher-Rössler A, Dazzan P, Chitnis X, Aston J, Drewe M, Regional gray matter volume abnormalities in the at risk mental state. Biol Psychiatry. 2007;61:1148–56. 17098213 10.1016/j.biopsych.2006.08.009

[44] Cannon TD, Chung Y, He G, Sun D, Jacobson A, van Erp TG, Progressive reduction in cortical thickness as psychosis develops: a multisite longitudinal neuroimaging study of youth at elevated clinical risk. Biol Psychiatry. 2015;77:147–57. 25034946 10.1016/j.biopsych.2014.05.023 PMC4264996

[45] Fornito A, Yung AR, Wood SJ, Phillips LJ, Nelson B, Cotton S, Anatomic abnormalities of the anterior cingulate cortex before psychosis onset: an MRI study of ultra-high-risk individuals. Biol Psychiatry. 2008;64:758–65. 18639238 10.1016/j.biopsych.2008.05.032

[46] Harris JM, Moorhead TW, Miller P, McIntosh AM, Bonnici HM, Owens DG, Increased prefrontal gyrification in a large high-risk cohort characterizes those who develop schizophrenia and reflects abnormal prefrontal development. Biol Psychiatry. 2007;62:722–9. 17509536 10.1016/j.biopsych.2006.11.027

[47] Walterfang M, Yung A, Wood AG, Reutens DC, Phillips L, Wood SJ, Corpus callosum shape alterations in individuals prior to the onset of psychosis. Schizophr Res. 2008;103:1–10. 18562178 10.1016/j.schres.2008.04.042

[48] Vita A, De Peri L, Deste G, Sacchetti E. Progressive loss of cortical gray matter in schizophrenia: a meta-analysis and meta-regression of longitudinal MRI studies. Transl Psychiatry. 2012;2:e190. 23168990 10.1038/tp.2012.116 PMC3565772

[49] Chan RC, Di X, McAlonan GM, Gong QY. Brain anatomical abnormalities in high-risk individuals, first-episode, and chronic schizophrenia: an activation likelihood estimation meta-analysis of illness progression. Schizophr Bull. 2011;37:177–88. 19633214 10.1093/schbul/sbp073 PMC3004195

[50] Sun D, Stuart GW, Jenkinson M, Wood SJ, McGorry PD, Velakoulis D, Brain surface contraction mapped in first-episode schizophrenia: a longitudinal magnetic resonance imaging study. Mol Psychiatry. 2009;14:976–86. 18607377 10.1038/mp.2008.34 PMC2773126

[51] Walterfang M, Wood AG, Reutens DC, Wood SJ, Chen J, Velakoulis D, Morphology of the corpus callosum at different stages of schizophrenia: cross-sectional study in first-episode and chronic illness. Br J Psychiatry. 2008;192:429–34. 18515892 10.1192/bjp.bp.107.041251

[52] Steen RG, Mull C, McClure R, Hamer RM, Lieberman JA. Brain volume in first-episode schizophrenia: systematic review and meta-analysis of magnetic resonance imaging studies. Br J Psychiatry. 2006;188:510–8. 16738340 10.1192/bjp.188.6.510

[53] Vita A, De Peri L, Silenzi C, Dieci M. Brain morphology in first-episode schizophrenia: a meta-analysis of quantitative magnetic resonance imaging studies. Schizophr Res. 2006;82:75–88. 16377156 10.1016/j.schres.2005.11.004

[54] Nakamura M, Salisbury DF, Hirayasu Y, Bouix S, Pohl KM, Yoshida T, Neocortical gray matter volume in first-episode schizophrenia and first-episode affective psychosis: a cross-sectional and longitudinal MRI study. Biol Psychiatry. 2007;62:773–83. 17586477 10.1016/j.biopsych.2007.03.030 PMC2782514

[55] Radua J, Borgwardt S, Crescini A, Mataix-Cols D, Meyer-Lindenberg A, McGuire PK, Multimodal meta-analysis of structural and functional brain changes in first episode psychosis and the effects of antipsychotic medication. Neurosci Biobehav Rev. 2012;36:2325–33. 22910680 10.1016/j.neubiorev.2012.07.012

[56] Fusar-Poli P, Smieskova R, Serafini G, Politi P, Borgwardt S. Neuroanatomical markers of genetic liability to psychosis and first episode psychosis: a voxelwise meta-analytical comparison. World J Biol Psychiatry. 2014;15:219–228. 22283467 10.3109/15622975.2011.630408

[57] De Peri L, Crescini A, Deste G, Fusar-Poli P, Sacchetti E, Vita A. Brain structural abnormalities at the onset of schizophrenia and bipolar disorder: a meta-analysis of controlled magnetic resonance imaging studies. Curr Pharm Des. 2012;18:486–94. 22239579 10.2174/138161212799316253

[58] Gur RE, Cowell P, Turetsky BI, Gallacher F, Cannon T, Bilker W, A follow-up magnetic resonance imaging study of schizophrenia. Relationship of neuroanatomical changes to clinical and neurobehavioral measures. Arch Gen Psychiatry. 1998;55:145–52. 9477928 10.1001/archpsyc.55.2.145

[59] Velakoulis D, Wood SJ, Wong MT, McGorry PD, Yung A, Phillips L, Hippocampal and amygdala volumes according to psychosis stage and diagnosis: a magnetic resonance imaging study of chronic schizophrenia, first-episode psychosis, and ultra-high-risk individuals. Arch Gen Psychiatry. 2006;63:139–49. 16461856 10.1001/archpsyc.63.2.139

[60] Adriano F, Caltagirone C, Spalletta G. Hippocampal volume reduction in first-episode and chronic schizophrenia: a review and meta-analysis. Neuroscientist. 2012;18:180–200. 21531988 10.1177/1073858410395147

[61] Vita A, de Peri L. Hippocampal and amygdala volume reductions in first-episode schizophrenia. Br J Psychiatry. 2007;190:271. 10.1192/bjp.190.3.271 17329755

[62] Andreasen NC, Nopoulos P, Magnotta V, Pierson R, Ziebell S, Ho BC. Progressive brain change in schizophrenia: a prospective longitudinal study of first-episode schizophrenia. Biol Psychiatry. 2011;70:672–9. 21784414 10.1016/j.biopsych.2011.05.017 PMC3496792

[63] DeLisi LE, Sakuma M, Tew W, Kushner M, Hoff AL, Grimson R. Schizophrenia as a chronic active brain process: a study of progressive brain structural change subsequent to the onset of schizophrenia. Psychiatry Res. 1997;74:129–40. 9255858 10.1016/s0925-4927(97)00012-7

[64] Moser DA, Doucet GE, Lee WH, Rasgon A, Krinsky H, Leibu E, Multivariate Associations Among Behavioral, Clinical, and Multimodal Imaging Phenotypes in Patients With Psychosis. JAMA Psychiatry. 2018;75:386–95. 29516092 10.1001/jamapsychiatry.2017.4741 PMC5875357

[65] Hulshoff Pol HE, Kahn RS. What happens after the first episode? A review of progressive brain changes in chronically ill patients with schizophrenia. Schizophr Bull. 2008;34:354–66. 18283048 10.1093/schbul/sbm168 PMC2632411

[66] Ellison-Wright I, Glahn DC, Laird AR, Thelen SM, Bullmore E. The anatomy of first-episode and chronic schizophrenia: an anatomical likelihood estimation meta-analysis. Am J Psychiatry. 2008;165:1015–23. 18381902 10.1176/appi.ajp.2008.07101562 PMC2873788

[67] Olabi B, Ellison-Wright I, McIntosh AM, Wood SJ, Bullmore E, Lawrie SM. Are there progressive brain changes in schizophrenia? A meta-analysis of structural magnetic resonance imaging studies. Biol Psychiatry. 2011;70:88–96. 21457946 10.1016/j.biopsych.2011.01.032

[68] Fusar-Poli P, Smieskova R, Kempton MJ, Ho BC, Andreasen NC, Borgwardt S. Progressive brain changes in schizophrenia related to antipsychotic treatment? A meta-analysis of longitudinal MRI studies. Neurosci Biobehav Rev. 2013;37:1680–91. 23769814 10.1016/j.neubiorev.2013.06.001 PMC3964856

[69] Davidson LL, Heinrichs RW. Quantification of frontal and temporal lobe brain-imaging findings in schizophrenia: a meta-analysis. Psychiatry Res. 2003;122:69–87. 12714172 10.1016/s0925-4927(02)00118-x

[70] Ellison-Wright I, Bullmore E. Anatomy of bipolar disorder and schizophrenia: a meta-analysis. Schizophr Res. 2010;117:1–12. 20071149 10.1016/j.schres.2009.12.022

[71] Nelson MD, Saykin AJ, Flashman LA, Riordan HJ. Hippocampal volume reduction in schizophrenia as assessed by magnetic resonance imaging: a meta-analytic study. Arch Gen Psychiatry. 1998;55:433–40. 9596046 10.1001/archpsyc.55.5.433

[72] Shepherd AM, Laurens KR, Matheson SL, Carr VJ, Green MJ. Systematic meta-review and quality assessment of the structural brain alterations in schizophrenia. Neurosci Biobehav Rev. 2012;36:1342–56. 22244985 10.1016/j.neubiorev.2011.12.015

[73] Haijma SV, Van Haren N, Cahn W, Koolschijn PC, Hulshoff Pol HE, Kahn RS. Brain volumes in schizophrenia: a meta-analysis in over 18 000 subjects. Schizophr Bull. 2013;39:1129–38. 23042112 10.1093/schbul/sbs118 PMC3756785

[74] van Erp TG, Hibar DP, Rasmussen JM, Glahn DC, Pearlson GD, Andreassen OA, Subcortical brain volume abnormalities in 2028 individuals with schizophrenia and 2540 healthy controls via the ENIGMA consortium. Mol Psychiatry. 2016;21:585. 26283641 10.1038/mp.2015.118 PMC5751698

[75] Shenton ME, Dickey CC, Frumin M, McCarley RW. A review of MRI findings in schizophrenia. Schizophr Res. 2001;49:1–52. 10.1016/s0920-9964(01)00163-3 PMC2812015 11343862

[76] Heinze K, Reniers RL, Nelson B, Yung AR, Lin A, Harrison BJ, Discrete alterations of brain network structural covariance in individuals at ultra-high risk for psychosis. Biol Psychiatry. 2015;77:989–96. 25524754 10.1016/j.biopsych.2014.10.023

[77] Li RR, Lyu HL, Liu F, Lian N, Wu RR, Zhao JP, Altered functional connectivity strength and its correlations with cognitive function in subjects with ultra-high risk for psychosis at rest. CNS Neurosci Ther. 2018;24(12):1140–8. doi: 10.1111/cns.12865 29691990 PMC6489739

[78] Li T, Wang Q, Zhang J, Rolls ET, Yang W, Palaniyappan L, Brain-Wide Analysis of Functional Connectivity in First-Episode and Chronic Stages of Schizophrenia. Schizophr Bull. 2017;43:436–48. 27445261 10.1093/schbul/sbw099 PMC5605268

[79] Frangou S. A systems neuroscience perspective of schizophrenia and bipolar disorder. Schizophr Bull. 2014;40:523–31. 24609453 10.1093/schbul/sbu017 PMC3984528

[80] Gong Q, Hu X, Pettersson-Yeo W, Xu X, Lui S, Crossley N, Network-Level Dysconnectivity in Drug-Naïve First-Episode Psychosis: Dissociating Transdiagnostic and Diagnosis-Specific Alterations. Neuropsychopharmacology. 2017;42:933–40. 27782128 10.1038/npp.2016.247 PMC5312071

[81] Ganella EP, Seguin C, Pantelis C, Whittle S, Baune BT, Olver J, Resting-state functional brain networks in first-episode psychosis: A 12-month follow-up study. Aust N Z J Psychiatry. 2018;52(9):864–75. doi: 10.1177/0004867418775833 29806483

[82] Li F, Lui S, Yao L, Hu J, Lv P, Huang X, Longitudinal Changes in Resting-State Cerebral Activity in Patients with First-Episode Schizophrenia: A 1-Year Follow-up Functional MR Imaging Study. Radiology. 2016;279:867–75. 27007945 10.1148/radiol.2015151334

[83] Baker JT, Holmes AJ, Masters GA, Yeo BT, Krienen F, Buckner RL, Disruption of cortical association networks in schizophrenia and psychotic bipolar disorder. JAMA Psychiatry. 2014;71:109–18. 24306091 10.1001/jamapsychiatry.2013.3469 PMC4435541

[84] Fusar-Poli P, Crossley N, Woolley J, Carletti F, Perez-Iglesias R, Broome M, White matter alterations related to P300 abnormalities in individuals at high risk for psychosis: an MRI-EEG study. J Psychiatry Neurosci. 2011;36:239–48. 21299920 10.1503/jpn.100083 PMC3120892

[85] Walterfang M, McGuire PK, Yung AR, Phillips LJ, Velakoulis D, Wood SJ, White matter volume changes in people who develop psychosis. Br J Psychiatry. 2008;193:210–5. 18757979 10.1192/bjp.bp.107.043463

[86] Mittal VA, Dean DJ, Bernard JA, Orr JM, Pelletier-Baldelli A, Carol EE, Neurological soft signs predict abnormal cerebellar-thalamic tract development and negative symptoms in adolescents at high risk for psychosis: a longitudinal perspective. Schizophr Bull. 2014;40:1204–15. 24375457 10.1093/schbul/sbt199 PMC4193696

[87] Di Biase MA, Cropley VL, Baune BT, Olver J, Amminger GP, Phassouliotis C, White matter connectivity disruptions in early and chronic schizophrenia. Psychol Med. 2017;47:2797–810. 28528586 10.1017/S0033291717001313

[88] Yao L, Lui S, Liao Y, Du MY, Hu N, Thomas JA, White matter deficits in first episode schizophrenia: an activation likelihood estimation meta-analysis. Prog Neuropsychopharmacol Biol Psychiatry. 2013;45:100–6. 23648972 10.1016/j.pnpbp.2013.04.019

[89] Lee SH, Kubicki M, Asami T, Seidman LJ, Goldstein JM, Mesholam-Gately RI, Extensive white matter abnormalities in patients with first-episode schizophrenia: a Diffusion Tensor Iimaging (DTI) study. Schizophr Res. 2013;143:231–8. 23290268 10.1016/j.schres.2012.11.029 PMC4354799

[90] Hamoda HM, Makhlouf AT, Fitzsimmons J, Rathi Y, Makris N, Mesholam-Gately RI, Abnormalities in thalamo-cortical connections in patients with first-episode schizophrenia: a two-tensor tractography study. Brain Imaging Behav. 2019;13(2):472–81. doi: 10.1007/s11682-018-9862-8 29667043 PMC6192863

[91] Di X, Chan RC, Gong QY. White matter reduction in patients with schizophrenia as revealed by voxel-based morphometry: an activation likelihood estimation meta-analysis. Prog Neuropsychopharmacol Biol Psychiatry. 2009;33:1390–4. 19744536 10.1016/j.pnpbp.2009.08.020

[92] Ellison-Wright I, Bullmore E. Meta-analysis of diffusion tensor imaging studies in schizophrenia. Schizophr Res. 2009;108:3–10. 19128945 10.1016/j.schres.2008.11.021

[93] McIntosh AM, Muñoz Maniega S, Lymer GK, McKirdy J, Hall J, Sussmann JE, White matter tractography in bipolar disorder and schizophrenia. Biol Psychiatry. 2008;64:1088–92. 18814861 10.1016/j.biopsych.2008.07.026

[94] Bartholomeusz CF, Cropley VL, Wannan C, Di Biase M, McGorry PD, Pantelis C. Structural neuroimaging across early-stage psychosis: Aberrations in neurobiological trajectories and implications for the staging model. Aust N Z J Psychiatry. 2017;51:455–76. 27733710 10.1177/0004867416670522

[95] Kane JM, Robinson DG, Schooler NR, Mueser KT, Penn DL, Rosenheck RA, Comprehensive Versus Usual Community Care for First-Episode Psychosis: 2-Year Outcomes From the NIMH RAISE Early Treatment Program. Am J Psychiatry. 2016;173:362–72. 26481174 10.1176/appi.ajp.2015.15050632 PMC4981493

[96] Bertelsen M, Jeppesen P, Petersen L, Thorup A, Øhlenschlaeger J, Le Quach P, Course of illness in a sample of 265 patients with first-episode psychosis--five-year follow-up of the Danish OPUS trial. Schizophr Res. 2009;107:173–8. 18945593 10.1016/j.schres.2008.09.018

[97] Levine SZ, Rabinowitz J, Ascher-Svanum H, Faries DE, Lawson AH. Extent of attaining and maintaining symptom remission by antipsychotic medication in the treatment of chronic schizophrenia: evidence from the CATIE study. Schizophr Res. 2011;133:42–6. 22000938 10.1016/j.schres.2011.09.018

[98] Namhcw R. From Discovery to Cure: Accelerating the development of new and personalized interventions for mental illness. Available from: https://www.nimh.nih.gov/about/advisory-boards-and-groups/namhc/reports/fromdiscoverytocure_103739.pdf. Accessed 2020 Feb 11.

[99] Fusar-Poli P, Howes OD, Allen P, Broome M, Valli I, Asselin MC, Abnormal frontostriatal interactions in people with prodromal signs of psychosis: a multimodal imaging study. Arch Gen Psychiatry. 2010;67:683–91. 20603449 10.1001/archgenpsychiatry.2010.77

[100] Witthaus H, Mendes U, Brüne M, Ozgürdal S, Bohner G, Gudlowski Y, Hippocampal subdivision and amygdalar volumes in patients in an at-risk mental state for schizophrenia. J Psychiatry Neurosci. 2010;35:33–40. 20040244 10.1503/jpn.090013 PMC2799502

[101] Mathalon DH, Sullivan EV, Lim KO, Pfefferbaum A. Progressive brain volume changes and the clinical course of schizophrenia in men: a longitudinal magnetic resonance imaging study. Arch Gen Psychiatry. 2001;58:148–57. 11177116 10.1001/archpsyc.58.2.148

[102] Mourao-Miranda J, Reinders AA, Rocha-Rego V, Lappin J, Rondina J, Morgan C, Individualized prediction of illness course at the first psychotic episode: a support vector machine MRI study. Psychol Med. 2012;42:1037–47. 22059690 10.1017/S0033291711002005 PMC3315786

[103] van Haren NE, Cahn W, Hulshoff Pol HE, Kahn RS. Schizophrenia as a progressive brain disease. Eur Psychiatry. 2008;23:245–54. 18513927 10.1016/j.eurpsy.2007.10.013

[104] de Castro-Manglano P, Mechelli A, Soutullo C, Landecho I, Gimenez-Amaya JM, Ortuño F, Structural brain abnormalities in first-episode psychosis: differences between affective psychoses and schizophrenia and relationship to clinical outcome. Bipolar Disord. 2011;13:545–55. 22017223 10.1111/j.1399-5618.2011.00953.x

[105] Dempster K, Norman R, Théberge J, Densmore M, Schaefer B, Williamson P. Cognitive performance is associated with gray matter decline in first-episode psychosis. Psychiatry Res Neuroimaging. 2017;264:46–51. 28458083 10.1016/j.pscychresns.2017.04.007

[106] Dickinson D, Harvey PD. Systemic hypotheses for generalized cognitive deficits in schizophrenia: a new take on an old problem. Schizophr Bull. 2009;35:403–14. 18689868 10.1093/schbul/sbn097 PMC2659304

[107] Wannan CMJ, Cropley VL, Chakravarty MM, Bousman C, Ganella EP, Bruggemann JM, Evidence for Network-Based Cortical Thickness Reductions in Schizophrenia. Am J Psychiatry. 2019;176:552–63. 31164006 10.1176/appi.ajp.2019.18040380

[108] Klauser P, Zhou J, Lim JK, Poh JS, Zheng H, Tng HY, Lack of Evidence for Regional Brain Volume or Cortical Thickness Abnormalities in Youths at Clinical High Risk for Psychosis: Findings From the Longitudinal Youth at Risk Study. Schizophr Bull. 2015;41:1285–93. 25745033 10.1093/schbul/sbv012 PMC4601700

[109] Lawrie SM, Whalley HC, Abukmeil SS, Kestelman JN, Miller P, Best JJ, Temporal lobe volume changes in people at high risk of schizophrenia with psychotic symptoms. Br J Psychiatry. 2002;181:138–43. 12151285 10.1017/s0007125000161860

[110] Zalesky A, Pantelis C, Cropley V, Fornito A, Cocchi L, McAdams H, Delayed Development of Brain Connectivity in Adolescents With Schizophrenia and Their Unaffected Siblings. JAMA Psychiatry. 2015;72:900–8. 26176706 10.1001/jamapsychiatry.2015.0226

[111] DeLisi LE, Stritzke P, Riordan H, Holan V, Boccio A, Kushner M, The timing of brain morphological changes in schizophrenia and their relationship to clinical outcome. Biol Psychiatry. 1992;31:241–54. 1547298 10.1016/0006-3223(92)90047-4

[112] Van Rheenen TE, Cropley V, Zalesky A, Bousman C, Wells R, Bruggemann J, Widespread Volumetric Reductions in Schizophrenia and Schizoaffective Patients Displaying Compromised Cognitive Abilities. Schizophr Bull. 2018;44:560–74. 28981831 10.1093/schbul/sbx109 PMC5890481

[113] Weinberg D, Lenroot R, Jacomb I, Allen K, Bruggemann J, Wells R, Cognitive Subtypes of Schizophrenia Characterized by Differential Brain Volumetric Reductions and Cognitive Decline. JAMA Psychiatry. 2016;73:1251–9. 27829096 10.1001/jamapsychiatry.2016.2925

[114] Woodward ND, Heckers S. Brain Structure in Neuropsychologically Defined Subgroups of Schizophrenia and Psychotic Bipolar Disorder. Schizophr Bull. 2015;41:1349–59. 25904725 10.1093/schbul/sbv048 PMC4601708

[115] Castro-Fornieles J, Bargalló N, Calvo A, Arango C, Baeza I, Gonzalez-Pinto A, Gray matter changes and cognitive predictors of 2-year follow-up abnormalities in early-onset first-episode psychosis. Eur Child Adolesc Psychiatry. 2018;27:113–26. 28707138 10.1007/s00787-017-1013-z

[116] First MB, Williams JBW, Karg RS, Spitzer RL. Structured Clinical Interview for DSM-5 - Research Version (SCID-5 for DSM-5, Research Version; SCID-5-RV). Arlington (VA, US): American Psychiatric Association; 2015

[117] Wechsler D. Wechsler Abberviated Scale of Intelligence, Second Edition (WASI-II) San Antonio (TX US): NCS Pearson; 2011

[118] Hodes RJ, Insel TR, Landis SC, NIH BFNR. The NIH toolbox: setting a standard for biomedical research. Neurology. 2013;80:S1. 10.1212/WNL.0b013e3182872e90 PMC3662338 23479536

[119] Hollingshead AB. Two Factor Index of Social Position. New Haven (CT, US): Yale University; 1957.

[120] Kay SR, Fiszbein A, Opler LA. The positive and negative syndrome scale (PANSS) for schizophrenia. Schizophr Bull. 1987;13:261–76. 3616518 10.1093/schbul/13.2.261

[121] Kring AM, Gur RE, Blanchard JJ, Horan WP, Reise SP. The Clinical Assessment Interview for Negative Symptoms (CAINS): final development and validation. Am J Psychiatry. 2013;170:165–72. 23377637 10.1176/appi.ajp.2012.12010109 PMC3785242

[122] Young RC, Biggs JT, Ziegler VE, Meyer DA. A rating scale for mania: reliability, validity and sensitivity. Br J Psychiatry. 1978;133:429–35. 728692 10.1192/bjp.133.5.429

[123] Montgomery SA, Asberg M. A new depression scale designed to be sensitive to change. Br J Psychiatry. 1979;134:382–9. 444788 10.1192/bjp.134.4.382

[124] Niv N, Cohen AN, Sullivan G, Young AS. The MIRECC version of the Global Assessment of Functioning scale: reliability and validity. Psychiatr Serv. 2007;58:529–35. 17412856 10.1176/ps.2007.58.4.529

[125] Seidman LJ, Meyer EC, Giuliano AJ, Breiter HC, Goldstein JM, Kremen WS, Auditory working memory impairments in individuals at familial high risk for schizophrenia. Neuropsychology. 2012;26:288–303. 22563872 10.1037/a0027970 PMC3539430

[126] Seidman LJ, Breiter HC, Goodman JM, Goldstein JM, Woodruff PW, O’Craven K, A functional magnetic resonance imaging study of auditory vigilance with low and high information processing demands. Neuropsychology. 1998;12:505–18. 9805320 10.1037//0894-4105.12.4.505

[127] Van Essen DC, Ugurbil K, Auerbach E, Barch D, Behrens TE, Bucholz R, The Human Connectome Project: a data acquisition perspective. Neuroimage. 2012;62:2222–31. 22366334 10.1016/j.neuroimage.2012.02.018 PMC3606888

[128] Galbraith S, Bowden J, Mander A. Accelerated longitudinal designs: An overview of modelling, power, costs and handling missing data. Stat Methods Med Res. 2017;26:374–98. 25147228 10.1177/0962280214547150 PMC5302089

[129] Fitzmaurice GM, Laird NM, Ware JH. Applied Longitudinal Analysis. 2nd ed. Hoboken (New Jersey, US): Wiley; 2011.

[130] Moerbeek M. The effects of the number of cohorts, degree of overlap among cohorts, and frequency of observation on power in accelerated longitudinal designs. Methodol Eur J Res Methods Behav Soci Sci. 2011;7:11–24.

[131] Eack SM, Hogarty GE, Cho RY, Prasad KM, Greenwald DP, Hogarty SS, Neuroprotective effects of cognitive enhancement therapy against gray matter loss in early schizophrenia: results from a 2-year randomized controlled trial. Arch Gen Psychiatry. 2010;67:674–82. 20439824 10.1001/archgenpsychiatry.2010.63 PMC3741671

